# The effect of transversus abdominis plane block application on postoperative analgesia quality and patient satisfaction after varicocele surgery: a randomized clinical trial

**DOI:** 10.2478/abm-2023-0053

**Published:** 2023-10-09

**Authors:** Ömer Faruk Boran, Aykut Urfalioglu, Mahmut Arslan, Fatih Mehmet Yazar, Bora Bilal, Yavuz Orak, Bülent Katı, Ayşe Azak Bozan

**Affiliations:** 1 Department of Anesthesiology and Reanimation, Sütçü Imam University School of Medicine, Kahramanmaraş 46000, Turkey; 2 Department of General Surgery, Sütçü Imam University School of Medicine, Kahramanmaraş 46000, Turkey; 3 Department of Urology, Harran University School of Medicine, Şanlıurfa, Kahramanmaraş 63000, Turkey; 4 Necip Fazıl State Hospital, Kahramanmaraş 46000, Turkey

**Keywords:** intrathecal injections, patient satisfaction, postoperative pain, spinal anesthesia, transversus abdominis plane block

## Abstract

**Background:**

Postoperative pain management is an important aspect of anesthesia care and multimodal analgesic techniques are generally recommended.

**Objective:**

To compare the effect of spinal anesthesia + transversus abdominis plane (TAP) block application on postoperative analgesia quality and patient satisfaction with spinal anesthesia + intrathecal morphine (ITM) application.

**Methods:**

A total of 70 patients were randomly separated into 2 groups as spinal anesthesia + TAP block (TAP block group, n = 34) and spinal anesthesia + ITM group (ITM group, n = 36). The groups were compared in respect of age, body mass index values, and visual analog scale (VAS) values at 0 h, 2 h, 6 h, 12 h, and 18 h, and patient satisfaction was scored by Quality Improvement in Postoperative Pain Management at 24 h.

**Results:**

The mean age of the patients was 32.52 ± 6.50 years in the TAP block group and 30.11 ± 5.62 years in the ITM group, with no statistically significant difference determined. There was no statistically significant difference in terms of VAS values at 0 h, 2 h, 6 h, 12 h, and 18 h. When the factors affecting postoperative patient satisfaction were evaluated, feeling fatigue after the surgery (r = −0.811, *P* = 0.001) and postoperative complications such as nausea, vomiting, and itching (r = −0.831, *P* = 0.001) were found to have a negative effect on patient satisfaction.

**Conclusion:**

Due to low complication rates, TAP block is an effective application for postoperative analgesia management in varicocele operations that increases patient satisfaction postoperatively.

Although there is still controversy on the issue of whether or not optimal pain control with minimal side effects affects postoperative surgical outcomes, there is a general consensus that optimal control of pain is associated with a shorter hospital stay and increased patient satisfaction [1, 2]. Postoperative pain management is an important aspect of anesthesia care and multimodal analgesic techniques are generally recommended [3].

Multimodal analgesia may include systemic or neuraxial opioids, centrally acting drugs such as anti-inflammatory agents, and paracetamol. Another method that can be used for this purpose is intrathecal (IT) morphine administration, which is known to provide excellent analgesia for the treatment of postoperative pain. However, there are some adverse effects that reduce patient satisfaction, such as nausea, vomiting, and itching in addition to delayed respiratory depression due to rostral expansion, which limits the use of this method [4, 5].

Recently, attention has been focused on the local anesthesia technique, known as transversus abdominis plane (TAP) block, which was first described by Kuppuvelumani et al. [6] and has become widely used to control postoperative pain [4, 7]. TAP block is defined as the administration of local anesthetic agents into the anatomic neurofascial cavity between the internal oblique and transversus abdominis muscle in the antero-lateral region of the abdomen to block the anterior branches of the thoracic intercostal (T7–T12) and first lumbar (L1) nerves [4, 7, 8]. This technique has been shown to be an effective analgesic regimen after many different surgical procedures such as cesarean section [9,10], abdominal surgery [11], and total abdominal hysterectomy [12]. However, in those studies, the analgesic efficacy was evaluated from the clinical perspective in general, and patient satisfaction has not been previously evaluated. In this study, evaluation was made of the effect of the TAP block method on postoperative patient satisfaction a day after the surgery, using the Quality Improvement in Postoperative Pain Management (QUIPS) test, which was first used in 2005, in addition to the parameters evaluated in previous studies, such as the postoperative analgesic consumption, and analgesic quality of the TAP block [13].

The study's primary aim was to determine the effect of spinal anesthesia + TAP block application on the reduction in the need for postoperative pain relief, and the secondary aim was to compare the effect of the increase in analgesia quality on patient satisfaction.

## Methods

Approval for the study was granted by the Kahramanmaras Sutcu Imam University Faculty of Medicine Clinical Research Ethical Committee (dated February 22, 2016; session No. 2016/04; decision No. 15), and all procedures were drafted in accordance with principles of the Helsinki Declaration. This blind, randomized, prospective, and controlled study was conducted in the urology and anesthesiology departments of Kahramanmaras Sutcu Imam University Hospital, between October 2016 and July 2018. The patients comprised in the study were informed about the methods before the procedure and written consent forms were obtained. A Clinical Trials number (ClinicalTrials.gov ID: NCT02983383) was also obtained before the study.

The study included a total of 70 consecutive patients, aged 18–45 years, evaluated as American Society of Anesthesiologists (ASA) I–II who underwent elective varicocele operations. Patients with a psychiatric disorder, usage of opioid analgesics, tricyclic antidepressants, or corticosteroids, hypersensitivity, severe systemic disorders such as cardiovascular, liver, and kidney disease, a drug and chronic pain killer addiction, anesthetic substance usage, alcohol addiction, pain syndrome, coagulopathy, infection in the surgical site, or recurrent varicocele were excluded from the study. The patients were randomly separated with the lottery method (specifically, the ball-pick method) into two groups, as the spinal anesthesia + TAP block group (TAP block, n = 34) and the spinal anesthesia + intrathecal morphine (ITM) group (ITM, n = 36).

### Anesthesia

All patients who received regional anesthesia were administered 0.02 mg/kg of midazolam IV sedation prior to the process. For spinal anesthesia, while the patients were sitting on the operating table, the area to be anesthetized was cleaned under sterile conditions and covered with a sterile perforated green cloth. With a median approach from the L4–L5 distance, the subarachnoid space was entered with a 26 G spinal needle. After the cerebrospinal fluid (CSF) flow was observed, the aperture of the needle was turned toward the sacral. By applying 10 mg of 0.5% hyperbaric bupivacaine, the head was lifted 30° when the patients were placed in the supine position after the patients were left in this position for 10 min.

In the ITM group, 0.05 mg/kg was applied. In the TAP block group, the procedure was performed under ultrasound (US) guidance using the method defined by the experienced anesthesiologists, Hebbart et al.[14], and then microscopic subinguinal varicocelectomy was performed. A MyLabTM 5 (Esaote) US device and LA 435 (6 MHz to −18 MHz) linear probes sterilized with antiseptic solution were used. Under US guidance, the probe was inserted between the costal margin and iliac crest, and then a 20-gauge 150 mm regional anesthesia needle (Stimuplex, B. Braun Melsungen AG) was advanced at the same level as the USG probe using the in-plane technique after visual confirmation of three muscle layers (from external to internal; inward external oblique, internal oblique, and transversus abdominis). The transversus abdominis muscle fascia was punctured and the needle tip was visualized between the internal oblique and transversus abdominis muscles. Following a 0.5–1 mL test dose, 20 mL of 0.25% bupivacaine (Marcaine 0.5%; AstraZeneca) was administered. In Group 2, 100 mcg of morphine was administrated as adjuvant to the spinal anesthesia.

### Postoperative procedures

The patients were evaluated for nausea, vomiting, itching, and respiratory depression at 0 h, 2 h, 6 h, 12 h, and 18 h, respectively.

Pain at rest and in motion (bodily and visceral; knee flexion) were measured with a 10 cm visual analog scale (VAS) pain score, from 0 = the absence of pain to 10 = the worst pain. Nausea severity was rated between 1–4 points, as 1 = absent, 2 = mild, 3 = moderate, and 4 = severe and vomiting.

Itching was rated between 1–4 points, as 1 = no pruritus, 2 = mild itching with no treatment required, 3 = moderate itching requiring treatment, and 4 = severe itching requiring treatment. A 24 h general evaluation was assessed at 24 h postoperatively using the QUIPS test.

A patient IV controlled pain monitoring device was used to control pain. The PCA protocol was a 25 mg tradamol bolus, with a locked time of 30 min, with no continuous infusion. The 4 h limit was 100 mg. The amount of analgesia and number of analgesic boluses used were recorded. Power analysis was applied to the study based on the results of previous studies.

Evaluation was made of all the patients in respect of age, gender, body mass index (BMI), duration of operation, ASA values, 0 h, 2 h, 6 h, 12 h, and 18 h VAS values, operated side, patient satisfaction, and anesthesia complications causing patient dissatisfaction such as nausea, vomiting, and itching, and the amount and time of first analgesic use.

### Sample size calculation

**Figure 1.**
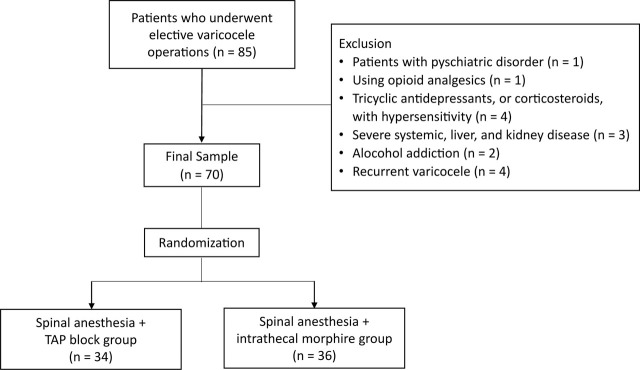
CONSORT diagram of studied patients.

The approximate sample size was calculated using the G*Power 3.1.9.4 analysis program (Heinrich-Heine-Universitat). We sought to investigate the analgesic efficiency of the USG-TAP block used for varicocele surgeries. The power analysis was based on the mean postoperative analgesia consumption. The sample size estimation was based on the postoperative tradamol requirements (control group: 309.6 ± 90.1; USG-TAP block: 234.0 ± 135.9) of a similar study performed by Kupiec et al. [15]. The sample size was calculated at a power of 85% and a significance level of 5%, and it was determined that it would be necessary to have approximately 34 patients per group to obtain significant statistical value. To be most conservative, a total of 70 patients were included in the research, with 34 patients in the treatment group and 36 patients in the control group (**Figure 1**).

### Statistical analysis

Data obtained in the study were analyzed statistically using SPSS 17.0 software (SPSS Inc.). Conformity of the data to normal distribution was tested using the Shapiro–Wilk test and variance homogeneity with the Levene's test. In the comparisons of two independent groups, the Mann–Whitney *U* test was used with the Monte Carlo simulation technique, and the independent-samples *T* test was used with bootstrap results. In the comparisons of categorical data, the Spearman's rank correlation and Fisher's exact tests were applied with the Monte Carlo simulation technique. Quantitative data were expressed as mean ± standard deviation (SD) values and median (range, minimum–maximum) values in the tables. Categorical data were expressed as number (n) and percentage (%). The VAS values were analyzed by a repeated measure ANOVA followed by the Bonferroni test for between-group comparisons. The data were analyzed at 95% confidence interval level and a *P* < 0.05 was accepted as statistically significant.

## Results

**Figure 2.**
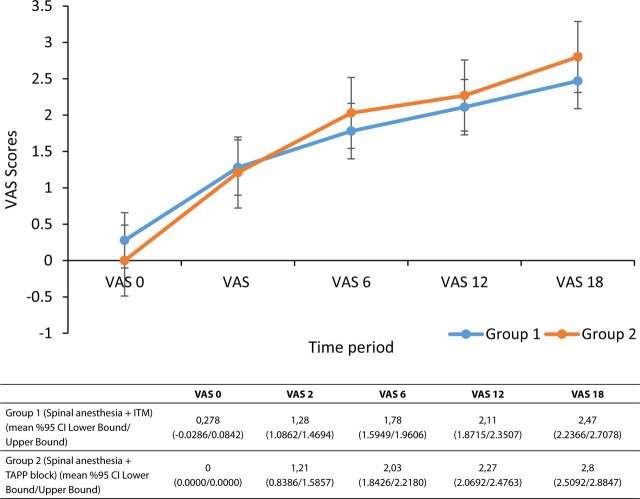
Postoperative pain values of the groups.

The mean age of the patients was 32.5 ± 6.5 years in the TAP block group (n = 34), and 30.1 ± 5.6 years (n = 36) in the spinal anesthesia group, and mean BMI values were 26.7 ± 3.9 and 23.1 ± 2.7, respectively. There was no statistical difference between the groups in respect of these parameters. Although the operation time in the spinal anesthesia group was shorter, the difference was not statistically significant (*P* = 0.174; **Table 1**). One patient in both groups avoided movement due to pain (*P* = 0.731), 4 patients in the TAP block group responded negatively, and 3 patients in the ITM group felt pain (*P* = 0.450). Tiredness after surgery was reported by 3 patients in the TAP block group and in 10 patients in the ITM group (*P* = 0.045). In our study, it was observed that VAS scores increased significantly in both groups over time [*F* (3, 37, 268) = 264.76; *P* < 0.001; η^2^ = 0.798], but the difference between the groups was not significant [*F* (3, 37, 268) = 1.40; *P* = 0.233; η^2^ = 0.021] (**Figure 2)**.

**Table 1.** Demographic data of the patients in the TAP block and ITM groups

	**TAP block group (n = 34)**	**ITM group (n = 36)**	***P***
Age (years)	32.52 ± 6.50	30.11 ± 5.62	0.105
BMI (kg/m^2^)	26.73 ± 3.96	23.11 ± −2.74	0.148
Operative time (min)	94.86 ± 38.27	82.79 ± 35.10	0.174
ASA Score			
1	16 (23.2%)	13 (18.9%)	0.213
2	17 (24.6%)	23 (33.3%)	
Side			
Right	0 (0.0%)	1 (1.45%)	0.584
Left	12 (17.4%)	11 (15.95%)	
Bilateral	22 (31.9%)	24 (34.8%)	
Rescue analgesia use (pts)	1 (1.45%)	1 (1.45%)	0.739
Postoperative mean pain	2.24 ± 0.71	2.27 ± 0.70	0.836
Postoperative max. pain	3.00 ± 1.08	2.77 ± 0.68	0.309
Postoperative min. pain	1.21 ± 1.05	1.27 ± 0.56	0.745
Patient satisfaction	12.30 ± 1.13	10.94 ± 3.06	0.019
Complication			
Nausea	0	4 (5.8%)	0.023
Itching	0	3 (4.35%)	
Nausea	0	2 (2.9%)	

Data are expressed as the mean ± SD, unless otherwise noted. Categorical data are expressed as n (number) and percentage (%). Independent *T* Test (Bootsrap), Whitney *U* test (Monte Carlo).

ASA, American Society of Anesthesiologists; BMI, body mass index; ITM, intrathecal morphine; SD, standard deviation; TAP, transversus abdominis plane.

There was a statistically significant difference between the groups in terms of patient satisfaction scores (*P* = 0.019). The results were significantly different in the spinal anesthesia group in terms of anesthesia complications (*P* = 0.023; **Table 1**).

When patients were evaluated in respect of patient-controlled pain monitoring, one patient in both groups used additional painkillers.

In the TAP block group, 1 patient who underwent bilateral varicocelectomy and bilateral TAP block first needed analgesia of 150 mg tramadol at 4 h postoperatively. The patient stated that he felt unilateral pain, which suggested unilateral unsuccessful block implementation. In the ITM group, analgesia requirement was found to be 75 mg tramadol at 10 h postoperatively. There was no statistically significant difference between the groups in terms of postoperative analgesic requirement (*P* = 0.751). When the patients were assessed in terms of factors affecting postoperative patient satisfaction, it was seen that feeling fatigue after surgery (r = −0.505, *P* < 0.001) and complications such as nausea, vomiting, and itching in the postoperative period (r = −0.831, *P* = 0.001) were found to have a negative effect on patient satisfaction. In the subgroup analysis, especially nausea (r = −0. 413, *P* = 0.001) and vomiting (r = −0. 355, *P* = 0.003) were found to have a greater effect than itching (r = −0. 295, *P* = 0.013) on patient satisfaction.

**Figure 3.**
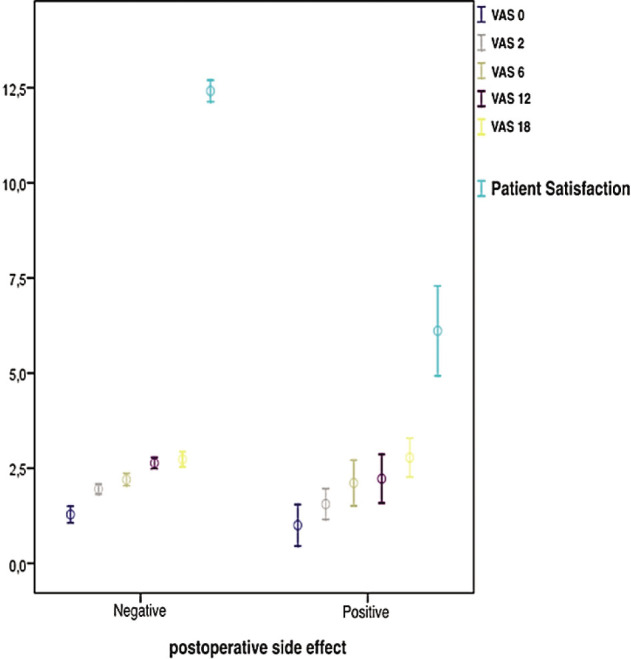
The relationships between visual analog scale values and patient satisfaction in patients with postoperative side effects.

When the patients were evaluated as patients with and without complications, there was no statistically significant difference in terms of VAS values at 0 h, 2 h, 6 h, 12 h, and 18 h (respectively, *P* = 0.342, *P* = 0.745, *P* = 0.054, *P* = 0.304, and *P* = 0.139). Patient satisfaction was found to be statistically significantly lower in patients with complications (nausea, vomiting, and itching) (*P* = 0.001). The VAS scores and patient satisfaction are shown in **Figure 3**.

**Figure 4.**
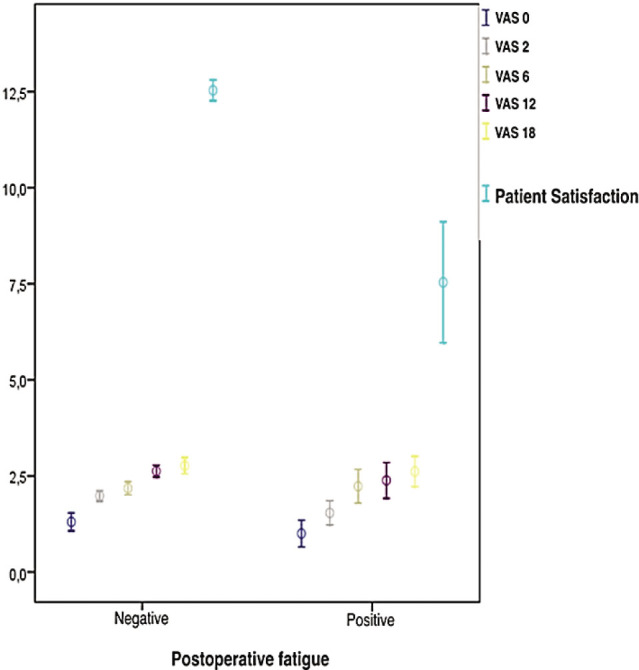
The relationships between postoperative fatigue, visual analog scale values, and patient satisfaction.

When the patients were grouped as patients with and without drowsiness-fatigue associated with patient satisfaction, the VAS values were lower in patients with drowsiness-fatigue, which was not statistically significant (all *P* > 0.05), and patient satisfaction was significantly higher in patients without drowsiness-fatigue (*P* = 0.001; **Figure 4**).

## Discussion

In this study, we evaluated the effects of TAP block application on patient satisfaction by comparing with ITM application in postoperative analgesia control in patients who underwent elective varicocelectomy operations. For this purpose, objective evaluation was made at 24 h postoperatively using the QUIPS test. The study results showed that postoperative fatigue and some complications such as nausea, vomiting, and pruritus were found to be highly associated with patient satisfaction, but not BMI.

To our knowledge, this is the first study to have evaluated the effect of TAP block on patient satisfaction. Although the effects of TAP block application on postoperative analgesic consumption and analgesic quality were evaluated in this study using a procedure similar to those employed in previous studies, there was additional evaluation of the effects of TAP block application on postoperative patient satisfaction on the 1st postoperative day using the QUIPS test, which was first used in 2005.

Varicocele is the most common pathology of male infertility, which can be seen in 15%–22% of the adult male population [16]. Although it is treated with laparoscopic or radiological (sclerotherapy or embolization) methods, open surgery is still the gold standard application [17]. In terms of anesthesia approaches, general, local, or regional anesthetic methods can be used for varicocele operations. Although local or regional approaches have some advantages such as low postoperative nausea and vomiting, suppression of stress response to surgical intervention, reduction of morbidity in high-risk patients, continuation of the analgesic effect in the postoperative period, and fast recovery without respiratory depression or loss of consciousness, there are also some disadvantages such as arterial vasodilatation, peripheral reflex vasoconstriction, bradycardia, and hypotension, which are specific to the cardiovascular system, and urinary retention [18]. In contrast, while there are some adverse effects of general anesthesia such as nausea, vomiting, pain, and respiratory depression, it has the advantages of minimal urinary retention, and better control of hemodynamic parameters both intraoperatively and postoperatively [19]. However, postoperative pain management, which determines patient satisfaction, is an important aspect of anesthetic care and the generally recommended multimodal analgesic techniques have been widely used in varicocele surgery in recent years.

There are many studies in literature that have evaluated the effect of TAP block on postoperative pain control after different surgical procedures or have compared TAP block or other multimodal analgesic methods [7,8,9,10,11,12].

There are also two different studies that have evaluated the efficacy of TAP block after varicocelectomy as in the current study. The efficacies of conventional spinal anesthesia and TAP block were compared in patients undergoing retroperitoneal varicocelectomy in the first study and it was reported that the anesthetic efficacy of the TAP block method was similar to that of spinal anesthesia. The VAS values of both methods were similar in the postoperative period [20]. In the other study, morphine and TAP block were compared in patients undergoing varicocelectomy with general anesthesia in terms of postoperative pain control and both methods were found to have similar effects on pain control, but TAP block application was found to be significantly superior in terms of some somatic effects such as nausea, vomiting, and itching [21].

In the present study, the QUIPS test was applied at 24 h postoperatively. The most important limitation of this test is that it provides only one measurement for pain and does not include the whole 24 h period [22]. To overcome this, VAS values were taken at 0 h, 6 h, 12 h, and 18 h to ensure that optimal evaluation of the pain process was performed. No difference in VAS values was determined between the groups (**Figure 2**). In addition, the pain values obtained throughout the first 24 h postoperative period were evaluated as mean pain value throughout 24 h, and maximum and minimum pain values. There were no statistically significant differences between the groups in terms of these values. It is known that respiratory functions are affected and the cough reflex is suppressed due to severe postoperative pain, which is particularly prone to complications such as atelectasis. As some authors have stated, optimal pain control is not only a factor that may shorten hospital stay but also affects surgical outcomes in the postoperative period by preventing some lung complications such as atelectasis [23, 24]. Pain felt on respiration or coughing in the postoperative period was reported by 11.8% of the TAP group and by 6.5% of the ITM group, but there was no significant difference between the groups (*P* = 0.450). In addition, none of the patients in the present study developed AC complication, postoperatively. Nevertheless, considering that the operation site chosen in the present study was the lower abdominal region, we infer that further studies are needed to evaluate the effect of TAP block on respiratory complications in abdominal surgery.

Although the ITM application is known to have highly effective analgesic efficacy, it has severe potential side effects [25,26,27]. Therefore, it is not preferred for postoperative analgesia, and analgesic regimens with a lower side-effect profile are generally preferred. In the present study, although the analgesic activity was good in the ITM group, patient satisfaction was found to be negatively affected in patients with morphine side effects. Correlation analysis revealed that the most effective factors on postoperative patient satisfaction were postoperative fatigue and dizziness (r = −0.811, *P* = 0.001). When the patients were separated as those with and without fatigue-drowsiness, the mean pain, maximum pain, and minimum pain values in patients with fatigue-drowsiness were lower than those of the patients without fatigue-drowsiness, but this difference was not statistically significant (*P* = 0.483, *P* = 0.175, *P* = 0.714).

Although there were fewer patients who did not feel fatigue-drowsiness postoperatively, the difference was not statistically significant (*P* = 0.483, *P* = 0.175, *P* = 0.714). Despite the lack of a statistically significant difference, the feeling of drowsiness was seen to be more disturbing to the patients in terms of satisfaction. Similar results were found for nausea, vomiting, and itching. In the subgroup analysis, especially nausea (r = −0.576, *P* = 0.001) and vomiting (r = −0.541, *P* = 0.001) had a greater effect on patient satisfaction than itching (r = −0.348, *P* = 0.003). Since we could not access data on this issue from the literature, we could not compare our study results with those of any previous study.

No vascular complications related to the TAP block developed in the current study. Therefore, the main limitation of the study was that no evaluation was made of the effect of such complications on patient satisfaction.

## Conclusion

In conclusion, due to low complication rates, TAP block is an effective application for postoperative analgesia management in varicocele operations that increases patient satisfaction postoperatively.

## References

[1] Schug SA (2011). 2011 – the global year against acute pain. Anaesth Intensive Care.

[2] Liu SS, Wu CL (2007). Effect of postoperative analgesia on major postoperative complications: a systematic update of the evidence. Anesth Analg.

[3] Mhuircheartaigh RJ, Moore RA, McQuay HJ (2009). Analysis of individual patient data from clinical trials: epidural morphine for postoperative pain. Br J Anaesth.

[4] Dewinter G, Van de Velde M, Fieuws S, D’Hoore A, Rex S (2014). Transversus abdominis plane block versus perioperative intravenous lidocaine versus patient-controlled intravenous morphine for postoperative pain control after laparoscopic colorectal surgery: study protocol for a prospective, randomized, double-blind controlled clinical trial. Trials.

[5] Marret E, Kurdi O, Zufferey P, Bonnet F (2005). Effects of nonsteroidal antiinflammatory drugs on patient-controlled analgesia morphine side effects: meta-analysis of randomized controlled trials. Anesthesiology.

[6] Kuppuvelumani P, Jaradi H, Delilkan A (1993). Abdominal nerve blockade for postoperative analgesia after caesarean section. Asia Oceania J Obstet Gynaecol.

[7] Abdallah FW, Halpern SH, Margarido CB (2012). Transversus abdominis plane block for postoperative analgesia after Caesarean delivery performed under spinal anaesthesia? A systematic review and meta-analysis. Br J Anaesth.

[8] Yu N, Long X, Lujan-Hernandez JR, Succar J, Xin X, Wang X (2014). Transversus abdominis-plane block versus local anesthetic wound infiltration in lower abdominal surgery: a systematic review and meta-analysis of randomized controlled trials. BMC Anesthesiol.

[9] Mishriky BM, George RB, Habib AS (2012). Transversus abdominis plane block for analgesia after Cesarean delivery: a systematic review and meta-analysis. Can J Anaesth.

[10] Johns N, O’Neill S, Ventham NT, Barron F, Brady RR, Daniel T (2012). Clinical effectiveness of transversus abdominis plane (TAP) block in abdominal surgery: a systematic review and meta-analysis. Colorectal Dis.

[11] Champaneria R, Shah L, Geoghegan J, Gupta JK, Daniels JP (2013). Analgesic effectiveness of transversus abdominis plane blocks after hysterectomy: a meta-analysis. Eur J Obstet Gynecol Reprod Biol.

[12] Kadihasanoglu M, Karaguzel E, Kacar CK, Arıkan MS, Yapici ME, Türkmen N (2012). Local or spinal anesthesia in subinguinal varicocelectomy: a prospective randomized trial. Urology.

[13] Sajadi H, Hosseini J, Farrahi F, Dadkhah F, Sepidarkish M, Sabbaghian M (2019). Varicocelectomy may improve results for sperm retrieval and pregnancy rate in non-obstructive azoospermic men. Int J Fertil Steril.

[14] Hebbard P, Fujiwara Y, Shibata Y, Royse C (2007). Ultrasound-guided transversus abdominis plane (TAP) block. Anaesth Intensive Care.

[15] Kupiec A, Zwierzchowski J, Kowal-Janicka J, Goździk W, Fuchs T, Pomorski M (2018). The analgesic efficiency of transversus abdominis plane (TAP) block after caesarean delivery. Ginekol Pol.

[16] Aklaya F (2008). Postoperative complications and nausea vomiting. Turkiye Klinikleri J Anest Reanim-Special Topics.

[17] Donati A, Mercuri G, Iuorio S, Scarcella M, Trabucchi C, Pelaia P, Pietropaoli P (2005). Hemodynamic modifications after subarachnoid anaesthesia evaluated with tranthoracic echocardiyography. Minerva Anestesiol.

[18] Raschke GF, Meissner W, Peisker A, Raschke GF, Meissner W, Peisker A (2018). Bilateral sagittal split osteotomy-parameters and correlations of postoperative pain management. Clin Oral Investig.

[19] Milone M, Di Minno MN, Musella M, Maietta P, Iacovazzo C, Milone F (2013). Ultrasound-guided transversus abdominis plane block for retroperitoneal varicocele repair: could it be an anesthesia method?. Updates Surg.

[20] Ömür D, Oğuzalp H, Kiraz HA, Ekin S, Alan C, Ersay AR, Hancı V (2016). The analgesic efficacy of ultrasound-guided transversus abdominis plane block on postoperative pain and morphine consumption in varicocelectomy. Saudi Med J.

[21] Peisker A, Meissner W, Raschke GF, Fahmy MD, Guentsch A, Schiller J, Schultze-Mosgau S (2018). Quality of postoperative pain management after maxillofacial fracture repair. J Craniofac Surg.

[22] Nguyen NT, Lee SL, Goldman C, Fleming N, Arango A, McFall R, Wolfe BM (2001). Comparison of pulmonary function and postoperative pain after laparoscopic versus open gastric bypass: a randomized trial. J Am Coll Surg.

[23] Shea RA, Brooks JA, Dayhoff NE, Keck J (2002). Pain intensity and postoperative pulmonary complications among the elderly after abdominal surgery. Heart Lung.

[24] Meylan N, Elia N, Lysakowski C, Tramer MR (2009). Benefit and risk of intrathecal morphine without local anaesthetic in patients undergoing major surgery: meta-analysis of randomized trials. Br J Anaesth.

[25] Baciarello M, Cornini A, Zasa M, Pedrona P, Scrofani G, Venuti FS, Fanelli G (2011). Intrathecal atropine to prevent postoperative nausea and vomiting after Cesarean section: a randomized, controlled trial. Minerva Anestesiol.

[26] Gwirtz KH, Young JV, Byers RS, Alley C, Levin K, Walker SG, Stoelting RK (1999). The safety and efficacy of intrathecal opioid analgesia for acute postoperative pain: seven years’ experience with 5,969 surgical patients at Indiana University Hospital. Anesth Analg.

[27] Liu X, Zhang J, Zhao H, Mei H, Lian Q, Shangguan W (2014). The effect of propofol on intrathecal morphine-induced pruritus and its mechanism. Anesth Analg.

